# Molecular Cloning, Expression and Transport Activity of SaNPF6.3/SaNRT1.1, a Novel Protein of the Low-Affinity Nitrate Transporter Family from the Euhalophyte *Suaeda altissima* (L.) Pall.

**DOI:** 10.3390/membranes13100845

**Published:** 2023-10-22

**Authors:** Olga I. Nedelyaeva, Dmitrii E. Khramov, Lyudmila A. Khalilova, Alena O. Konoshenkova, Anastasia V. Ryabova, Larissa G. Popova, Vadim S. Volkov, Yurii V. Balnokin

**Affiliations:** 1 K.A. Timiryazev Institute of Plant Physiology, Russian Academy of Sciences, Moscow 127276, Russia; khramov.de@yandex.ru (D.E.K.); lhalilova@mail.ru (L.A.K.); alenakonoshenkova@gmail.com (A.O.K.); lora_gp@mail.ru (L.G.P.); balnokin@mail.ru (Y.V.B.); 2 Prokhorov General Physics Institute of the Russian Academy of Sciences, Moscow 119991, Russia; nastya.ryabova@nsc.gpi.ru

**Keywords:** anion transporter, euhalophyte, NPF/NRT/PTR family, *Suaeda altissima*

## Abstract

The *SaNPF6.3* gene, a putative ortholog of the dual-affinity nitrate (NO_3_^−^) transporter gene *AtNPF6.3*/*AtNRT1.1* from *Arabidopsis thaliana*, was cloned from the euhalophyte *Suaeda altissima*. The nitrate transporting activity of SaNPF6.3 was studied by heterologous expression of the gene in the yeast *Hansenula* (*Ogataea*) *polymorpha* mutant strain Δ*ynt1* lacking the original nitrate transporter. Expression of *SaNPF6.3* in Δ*ynt1* cells rescued their ability to grow on the selective medium in the presence of nitrate and absorb nitrate from this medium. Confocal laser microscopy of the yeast cells expressing the fused protein GFP-SaNPF6.3 revealed GFP (green fluorescent protein) fluorescence localized predominantly in the cytoplasm and/or vacuoles. Apparently, in the heterologous expression system used, only a relatively small fraction of the GFP-SaNPF6.3 reached the plasma membrane of yeast cells. In *S. altissima* plants grown in media with either low (0.5 mM) or high (15 mM) NO_3_^−^; concentrations, *SaNPF6.3* was expressed at various ontogenetic stages in different organs, with the highest expression levels in roots, pointing to an important role of SaNPF6.3 in nitrate uptake. *SaNPF6.3* expression was induced in roots of nitrate-deprived plants in response to raising the nitrate concentration in the medium and was suppressed when the plants were transferred from sufficient nitrate to the lower concentration. When NaCl concentration in the nutrient solution was elevated, the *SaNPF6.3* transcript abundance in the roots increased at the low nitrate concentration and decreased at the high one. We also determined nitrate and chloride concentrations in the xylem sap excreted by detached *S. altissima* roots as a function of their concentrations in the root medium. Based on a linear increase in Cl^−^ concentrations in the xylem exudate as the external Cl^−^ concentration increased and the results of *SaNPF6.3* expression experiments, we hypothesize that SaNPF6.3 is involved in chloride transport along with nitrate transport in *S. altissima* plants.

## 1. Introduction

Nitrate (NO_3_^−^) is the main source of nitrogen, the important biogenic element for terrestrial plants [1,2]. Nitrate concentrations in soils vary widely and are often in the micromolar range, limiting plant growth [3]. Both low-affinity (LATS) and high-affinity (HATS) nitrate transport systems operate in plants, participating in NO_3_^−^ uptake by roots, translocation to shoots and allocation of N among tissues [1,3,4,5,6,7,8,9,10].

The *AtNPF6.3* (*CHL1*/*NRT1.1*) gene from *Arabidopsis thaliana* was the first to be cloned among the genes of the NO_3_^−^ transporting proteins [11]. The study of Tsay and coworkers [11] initiated identification of multiple homologous proteins, now grouped into a large family of low-affinity nitrate transporters: the NITRATE TRANSPORTER 1/PEPTIDE TRANSPORTER FAMILY (NPF/NRT1/PTR). There are 53 members of this family in *A. thaliana* [10]. NPF proteins exhibit different substrate specificity in plants. They transport not only nitrate but also other substances, including nitrite, peptides, amino acids, glucosinolates, auxin, abscisic acid and gibberellins [10,12]. In spite of belonging to the family of low-affinity transporters, AtNPF6.3 is involved in NO_3_^−^ absorption by roots in both a low-affinity and a high-affinity mode, depending on the external nitrate concentration. AtNPF6.3 displays biphasic Michaelis–Menten saturation kinetics as a HATS (Km ≈ 50 µM) at external nitrate concentrations less than 0.2–0.5 mM and as a LATS (Km ≈ 4 mM) at higher nitrate concentrations [7,13]. Switching between low-affinity and high-affinity modes is regulated by the phosphorylation status of Thr101 residue at the N-terminus in AtNPF6.3 [13,14]. The investigation of AtNPF6.3 crystal structure gave rise to the hypothesis that the switching mechanism of the dual-affinity nitrate transporter is based on a coupling–decoupling of homo-dimers of this protein. AtNPF6.3 in an unphosphorylated, structurally coupled state (dimer), functions as a low-affinity transporter. Phosphorylation of Thr101 uncouples the dimers and shifts the protein to a high-affinity state [15,16]. Using the *Xenopus* oocyte expression system, AtNPF6.3 was shown to be an electrogenic proton-coupled nitrate transporter, suggesting symport stoichiometry of H^+^/NO_3_^−^ > 1 [7,17].

Apart from transport functions, AtNPF6.3 serves as a nitrate sensor involved in the control of nitrate assimilation and developmental processes, including the regulation of expression of nitrate-related genes, root system development and breaking seed dormancy [10,12,18,19,20,21,22]. *A. thaliana NPF6.3* homologs have been identified in different plant species including *Brassica napus* (*BnNPF6.3/NRT1.1B/NRT1.2*) [23], *Oryza sativa* (*OsNPF6.3–6.5/NRT1.1A-C*) [12,24,25], *Zea mays* (*ZmNPF6.4/NRT1.1A, ZmNPF6.6/NRT1.1B, ZmNRT1.1C-D*) [24,25,26], *Medicago truncatula (MtNPF6.8/NRT1.3*) [27] and *Sorghum bicolor* (*SbNPF6.5/NRT1.1B*) [28]. Transport functions of the AtNPF6.3 homologs have been investigated using the *Xenopus* oocyte expression system. MtNPF6.8/NRT1.3, like AtNPF6.3, demonstrated dual-affinity kinetics with high (Km = 41.6 µM) and low (Km = 7.2 mM) nitrate affinity [27]. Some homologs demonstrated functional divergence from AtNPF6.3. For example, the Km for NO_3_^−^ uptake by BnNPF6.3/NRT1.2 was in the low-affinity range and grew from 4 to 14 mM as the membrane voltage was changed from −40 mV to −180 mV [23]. By contrast, *OsNPF6.4/NRT1.1A* displayed only low-affinity nitrate kinetics with a Km of 9 mM [29].

Salinization reduces nitrate availability to plants through competition between NO_3_^−^ and Cl^−^ for anionic transporters [30,31,32,33]; this could be crucial, especially for those plants growing in soils with low (micromolar) nitrate concentrations. However, few studies have investigated the role of AtNPF6.3 homologs in chloride transport. Using *Xenopus* oocytes, voltage clamp, ^36^Cl^−^, and ^15^N uptake techniques, Wen with coworkers [26] investigated anion transport by two AtNPF6.3 homologs from *Z. mays*, ZmNPF6.4/NRT1.1A and ZmNPF6.6/NRT1.1B. In the absence of NaCl, ZmNPF6.4 functioned as a low-affinity nitrate transporter with efflux activity, while ZmNPF6.6 demonstrated nitrate transporting activity with high-affinity kinetics (Km = 210 µM). However, under added Cl^−^, both proteins transported Cl^−^, ZmNPF6.4/NRT1.1A with high-affinity saturation kinetics (Km = 390 µM) and ZmNPF6.6/NRT1.1B with linear kinetics, in a wide range of external Cl^−^ concentrations. Higher uptake at acidic pH, compared to weakly alkaline pH, indicated that both transporters operated as Cl^−^/H^+^ symporters. Competition between NO_3_^−^ and Cl^−^ was shown for both transporters, with ZmNPF6.4 exhibiting Cl^−^ selectivity, while ZmNPF6.6 was selective for NO_3_^−^. The authors attributed the differences in the transport functions of the two *Z. mays* proteins, NPF6.4/NRT1.1A and NPF6.6/NRT1.1B, to different key polar residues which are involved in anion binding in the anion conducting tunnel [15,16]. The residue responsible for nitrate binding is presented by histidine (His 356) in ZmNPF6.6, while some other residue or domain, which remains to be identified, is involved in binding chloride in ZmNPF6.4 [26].

In other experiments, *A. thaliana*, *O. sativa* and *Nicotiana benthamiana* plants grown in a nutrient solution with NH_4_^+^ as a nitrogen source were found to be hypersensitive to NaCl stress [33]. For *AtNPF6.3* mutant plants, the authors showed that the salt hypersensitivity was a result of Cl^−^ overaccumulation due to AtNPF6.3 Cl^−^ transport activity, which was up-regulated by NH_4_^+^; salt stress hypersensitivity was alleviated in the presence of competing NO_3_^−^ anion.

Under saline conditions, halophytes, plants inhabiting saline soils, are suggested to regulate the absorption of nutrients more efficiently than glycophytes, plants that are not salt-tolerant [32,34,35,36]. However, available information on anion-transporting proteins of halophytes is scarce. Gene cloning and elucidation of functional features of anion transporters from halophytes are important for unraveling the mechanisms of plant salinity tolerance and improving crop salt tolerance by genetic engineering (reviewed by [37,38,39,40]).

Here, we describe cloning *SaNPF6.3*, a putative ortholog of *A. thaliana NPF6.3*/*NRT1.1*, from the euhalophyte *Suaeda altissima*. The genus *Suaeda* belongs to the Amaranthaceae (Chenopodiaceae), many members of which inhabit highly saline soils and are characterized by extreme salt tolerance [41,42]. *S. altissima* is one of the most salt tolerant plants and is able to perform its life cycle at NaCl concentrations up to 1M [43]. The ability of SaNPF6.3 to transport nitrate was examined by functional complementation analysis in the mutant strain Δ*ynt1* of the yeast *Hansenula* (*Ogatae*) *polymorpha*. *H. polymorpha* is a suitable model organism to study nitrate assimilation pathways in plants since it is able to take up and metabolize nitrate as the only nitrogen source [44,45,46,47,48]. Gene *YNT1* (yeast nitrate transporter 1) encodes the only high-affinity nitrate transporter in *H. polymorpha*. In the mutant strain Δ*ynt1*, the gene *YNT1* has been deleted. The nitrate transporting activity of SaNPF6.3 was also validated by checking the ability of Δ*ynt1* expressing *SaNPF6.3* to absorb NO_3_^−^ from nitrate-containing media. The relative *SaNPF6.3* transcript abundance in *S. altissima* organs was measured for plants grown at various nitrate and chloride concentrations in nutrient solutions. The changes in *SaNPF6.3* expression which were induced by changing the nitrate availability and salinity in the medium were also studied.

## 2. Material and Methods

### 2.1. Plant Material

Seeds of *Suaeda altissima* (L.) Pall. were collected from plants growing in the wild on the shores of the salt lake Elton, located in the Volgograd region in Russia. Seeds were stratified at +4 °C over 3 days and then germinated in wet sand. Seed germination and further plant growth were carried out in a growth chamber under controlled conditions at 24 °C and relative humidity of 60–70%. The plants were illuminated with high pressure sodium lamps DNaZ_400 (Reflux, Novocherkassk, Russia) at a light flux of 300 µmol photons m^−2^ s^−1^ and photoperiod of 16 h, with an 8 h dark period. Fourteen days after germination, the seedlings were transplanted to an aerated Robinson and Downton nutrient solution (NS) [49] in 3-L opaque glass containers (5 plants per container) with low (0.5 mM) or high (15 mM) nitrate concentrations. Plants were grown hydroponically under the same environmental conditions until they were 45 days old, when they were used in most experiments. For total RNA extraction, organs of *S. altissima* plants (roots, leaves, stems, flowers) were sampled (approximately 1 g fresh weight of each sample) and frozen in liquid nitrogen for further use.

To study the long-term salinity effect on the expression of *SaNPF6.3* in *Suaeda* organs, NaCl was applied to the NS after 7 d. To avoid salt shock, NaCl was added gradually in increments of 50 or 100 mM per day, up to the final concentrations of 250 or 750 mM; no NaCl was added to the NS for control plants. To study the effect of salt shock on the expression of *SaNPF6.3*, NaCl was raised to 250 mM in a single addition. *SaNPF6.3* transcript levels were determined after 31 d for both the long-term NaCl treatment and for the NaCl shock, when the plants had reached 45 d of age. In order to investigate the effects of changes in nitrite and ammonium ions on the expression of *SaNPF6.3*, KNO_2_ and (NH_4_)_2_SO_4_ were added to the NS at a final concentration of 5 mM after 31 d of plant growth in NS, with both a low (0.5 mM) or a high (15 mM) nitrate concentration. The effects of an increase in nitrate concentration in the NS from 0.5 mM up to 5 mM and the transfer of plants grown at 15 mM nitrate to the non-nitrate medium were also studied using plants of the same age. A comparative assessment of *SaNPF6.3* expression levels in different organs of *S. altissima* was performed for 21- and 45-day-old plants (7 and 31 days of growth in hydroponics, respectively) in roots, stems and leaves and for 60-day-old plants (at 46 day of growth in hydroponics) in flowers.

To obtain xylem exudates, the shoots of 55-day-old *S. altissima* plants grown under standard conditions in the NS medium but supplemented with nitrate and chloride at various concentrations (the ion concentrations are indicated in the corresponding figure), were cut off 2 cm above the root collar. Silicone tubes were placed on the stem stumps, and the exudates were collected in the tubes for 48 h at 24 °C. In the experiments where the dependence of NO_3_^−^ concentration in the xylem exudate on NO_3_^−^ concentration in the medium was determined, the plants were grown in the presence of 100 mM NaCl in NS. Two days before the exudate collection, the plants were transferred to modified NS, in which 1 mM KH_2_PO_4_ and 4 mM Ca(NO_3_)_2_ were replaced by 1 mM NaH_2_PO_4_ and 4 mM CaCl_2_, respectively, while KNO_3_ was added in various concentrations. In order to investigate the dependence of Cl^−^ concentration in the xylem exudate on Cl^−^ concentration in the medium, NaCl was applied to the NS gradually after 7 days of plant growth in hydroponics without NaCl, as indicated above.

### 2.2. Yeast Strain and Vectors Used in the Study

Methylotrophic yeast (*Hansenula polymorpha*) double auxotrophic strains DL-1 (*leu2 ura3* genotype) (wild-type strain, WT strain) and yeast integrative vectors pCCUR2 and pCHLX were used in this study. The strain DL-1 (*leu2 ura3*) was transformed with plasmids pCCUR2 and pCHLX carrying the URA and LEU genes, respectively, to ensure the growth of the yeast strains without additional nitrogen sources, leucine and uracil, when performing complementation tests. Plasmids pCCUR2 and pCHLX were kindly provided by Dr. Michael Agafonov (Federal Research Center “Fundamentals of Biotechnology”, Russian Academy of Sciences, Moscow, Russia). Yeast cells were transformed by the lithium method [50] or by electroporation [51] using an Eppendorf device (Eppendorf, Framingham, MA, USA).

### 2.3. Extraction of Total RNA from Plant Material and the First-Strand cDNA Synthesis

Total RNA from *S.altissima* plant organs was isolated by the hot phenolic method [52] and used as a template for the total first-strand cDNA synthesis. For amplification of the 3′- and 5′- ends of the *SaNPF6.3* transcript by the Step-Out RACE method, the first-strand cDNA was synthesized on the total RNA template, isolated from *Suaeda* roots, using MINT revertase (Evrogen, Moscow, Russia). Full-length cDNA of *SaNPF6.3* gene was also amplified on the total RNA template, isolated from *Suaeda* roots. To obtain full-length cDNA of *SaNPF6.3* and quantify the representation of the gene transcripts in *S. altissima* organs, first-strand cDNA synthesis was performed on total RNA templates using (dT)15 primer and MMLV revertase (Evrogen, Moscow, Russia).

### 2.4. Primer Design

For qPCR-RT experiments, primers were designed using Light Cycler 2.0 Probe Design software (https://lifescience.roche.com/, accessed on 4 July 2021). In other cases, primer Blast software, version 4.1.0 (https://www.ncbi.nlm.nih.gov/tools/primer-blast/, accessed on 4 July 2021) was used for the primer design. All primers used are listed in Table S1.

### 2.5. Identification of the Full Length SaNPF6.3 Coding Sequence

Partial coding sequence (the middle fragment) of the *SaNPF6.3* gene was obtained by us previously (GenBank ID: MK580125.1) [53]. Here, based on this sequence, the forward and reverse primer sets were designed for amplification of the 5′- and 3′-end sequences of *SaNPF6.3* cDNA. The 5′- and 3′-end sequences of *SaNPF6.3* cDNA were determined by the Step-Out RACE technology (kit #SKS03, Evrogen, Moscow, Russia). The cDNA fragments were amplified on the total cDNA template using Encyclo DNA polymerase (#PK002, Evrogen, Moscow, Russia). The 5′-sequence of *SaNPF6.3* cDNA (1152 bp) was amplified with primers SaNPF6.3_r (round 1) and SaNPF6.3_5′RACE_R1 (round 2). The 3′-sequence of *SaNPF6.3* cDNA (722 bp) was amplified with SaNPF6.3_f (round 1) and SaNPF6.3_F1 (round 2). All amplicons obtained, including also the middle cDNA fragment amplified from the cDNA template with a primer pair SaNPF6.3_f and SaNPF6.3_r, were cloned into pAL2-T vector (Evrogen, Moscow, Russia) for replication in *E. coli* cells and the following sequencing. Subsequently, the partial sequences (middle fragment, 5′- and 3′- ends of *SaNPF6.3* cDNA) were assembled in silico using SnapGene software 5.0.8 (https://www.snapgene.com/snapgene-viewer (accessed on 29 July 2023)). The resulting coding sequence of 1788 bp contained open reading frame (ORF) for the protein of 596 aa. This sequence was used for the design of primers for the amplification of full-length *SaNPF6.3* cDNA on the total first-strand cDNA template.

### 2.6. Cloning of the Full-Length SaNPF6.3 cDNA in the Yeast Vector pCHLX

The full-length coding sequence of *SaNPF6.3* was amplified from the total first-strand cDNA using primer pairs SaNPF6.3_Gib_F and SaNPF6.3_Gib_R. The full-length *SaNPF6.3* cDNA was cloned into the yeast integrative vector pCHLX [54] under the control of the inducible nitrate reductase promoter *pYNR1* and terminator *tYNR1* of *H. polymorpha*. Promoter *pYNR1* and terminator *tYNR1* sequences were amplified from the *H. polymorpha* genomic DNA template using primer pairs pYNR1_F and pYNR1_R, and tYNR1_F and tYNR1_R. The first 10 cycles of amplification of the promoter, terminator and gene coding sequences were performed using Encyclo polymerase (No. PK002, Evrogen, Moscow, Russia); the next 25 cycles were performed using CloneAmp HiFi PCR Premix kit (No. 639298, Clontech, Mountain View, CA, USA). The pCHLX vector was linearized in the Hind III and EcoRI restriction sites and ligated with the synthesized *pYNR1*, *tYNR1* and *SaNPF6.3* sequences using a Gibson assembly kit (No. E5510, SkyGen, NEB, Ipswich, MA, USA) to produce the pCHLX-*pYNR1*-*SaNPF6.3*-*tYNR1* construct (further denoted as pCHLX-*SaNPF6.3*). The cloned sequences were verified by sequencing. The *SaNPF6.3* cDNA sequence (1788 bp) was deposited in GenBank (GenBank ID: OQ330855).

The pCHLX-*GFP-SaNPF6.3* construct was also obtained for the expression of SaNPF6.3 fused at the N-terminus with green fluorescent protein (GFP) in yeast cells. To achieve this, three fragments were ligated in one reaction: (1) GFP coding sequence amplified from pTR vector [55] with pYNR1_GFP_F and TEV_GFP_R primers (2) *SaNPF6.3* coding sequence amplified from the first cDNA strand with TEV_SaNPF6.3_F and SaNPF6.3_Gib_R primers; (3) plasmid pCHLX linearized by inverted PCR from the pCHLX-* SaNPF6.3* construct with the pYNR1_R3 and tYNR1_F primer pair. Ligation was carried out using Gibson assembly kit (NEB, Ipswich, MA, USA).

### 2.7. Cloning of AtNPF6.3 Coding Sequence in the Yeast Vector pCHLX

To control the functional complementation of the *H. polymorpha* mutant Δ*ynt1*, the mutant was transformed by the construct carrying the known nitrate transporter gene *AtNPF6.3* (GenBank ID: NP_563899.1) from *Arabidopsis*. The coding sequence of *AtNPF6.3* was amplified from the *Arabidopsis* total first-strand cDNA, using primer pairs *AtNPF6.3_*Gib_F and *AtNPF6.3*_Gib_R. The total first-strand cDNA was synthesized on the total RNA template, isolated from roots of adult *Arabidopsis* plants. Like *SaNPF6.3* cDNA, full-length *AtNPF6.3* coding sequence was cloned into the vector pCHLX [54] under the control of the inducible nitrate reductase promoter *pYNR1* and terminator *tYNR1* of *H. polymorpha*. For ligation with *AtNPF6.3*, nitrate reductase promoter and terminator sequences were amplified from the *H. polymorpha* genomic DNA template using primer pairs pYNR1_F and pYNR1_R2, and tYNR1_F2 and tYNR1_R. All stages of the amplification and ligation of the sequences were similar to how it was carried out during the amplification and ligation of the sequences in the case of *SaNPF6.3* cloning. The construct pCHLX-*pYNR1-AtNPF6.3-tYNR1* (pCHLX-*AtNPF6.3*) was obtained for the transformation of mutant Δ*ynt1* cells.

### 2.8. Implementation of Amino Acid Substitutions in the Amino Acid Sequence of the SaNPF6.3 Protein

To obtain point amino acid substitutions of Tyr358His, Thr106Asp and Thr106Ala in the SaNPF6.3 protein sequence, the pCHLX-*pYNR1-SaNPF6.3-tYNR1* construct was linearized by inverse PCR with primer pairs SaNPF6.3_1074_F and SaNPF6.3_Tyr358His_R, SaNPF6.3_318_F and SaNPF6.3_Thr106Asp_R, and SaNPF6.3_318_F and SaNPF6.3_Thr106Ala_R, respectively, using a ready-to-use CloneAmp HiFi PCR Premix kit (TakaraBio, San Jose, CA, USA). The resulting linear forms of PCR products with corresponding nucleotide substitutions were converted into circular forms. For this purpose, the 5′-ends of the linearized plasmid DNA were phosphorylated using T4-polynucleotide kinase. The reaction mixture for the phosphorylation, containing T4 DNA-ligase buffer, 250 ng linearized DNA and 10 U T4-polynucleotid kinase (SibEnzyme, Novosibirsk, Russia), was incubated for 15 min at 37 °C. The phosphorylated product was ligated by adding 2.5 U T4 ligase (Fermentas, Thermo Fisher Scientific, Waltham, MA, USA) to the reaction mixture and incubating the mixture for 16–18 h at room temperature. The ligase was inactivated by incubation of the mixture at 65 °C for 15 min. Plasmid pCHLX-*SaNPF6.3*, which served as a template for amplification, was removed from the mixture by treatment with methyl-dependent DNA endonuclease Mal I (SibEnzyme, Novosibirsk, Russia). The mixture was purified from proteins by treatment with phenol-chloroform, and plasmid DNA was resuspended in mQH_2_O for subsequent transformation of competent *E. coli* cells.

### 2.9. Production of a Deletion Mutant Δynt1 of H. polymorpha

A mutant with a deleted *YNT1* gene (GenBank ID: CP080316.1), *ynt1::BleoR(ZeoR)*, was derived from the wild-type strain DL-1 (*leu2*, *ura3*) of *H. polymorpha* through homologous recombination by an “one-step gene disruption” method [56]. To carry out the knockout of the gene *YNT1*, the zeocin resistance gene (*ZeoR*) was incorporated into the *YNT1* locus through homologous recombination at the *YNT1* gene sites [44]. For this purpose, PCR fragments corresponding to “left” and “right” flanking parts of the *YNT1* gene and the zeocin resistance gene *ZeoR* amplified from the pVR2 vector [57] were cloned into the bacterial vector pBlueScript KS(II)+ using Gibson assembly kit (NEB, Ipswich, MA, USA). “Left” and “Right” fragments of the *YNT1* gene and the gene *ZeoR* were amplified using primer pairs ZeoYNTRfl_F and pBlueYNTRfl_R, pBlueYNTLfl_F and YNTLflZeo_R, and ZeoCas1 and ZeoCas2, respectively. From the resulting construct, pBlueScript KS(II)+ *YNT1_L-ZeoR- YNT1_R*, a fragment of *YNT1_L-ZeoR- YNT1_R,* was excised by Hind III and Pst I restriction endonucleases (SibEnzym, Novosibirsk, Russia) and transferred into DL1 cells. The presence of the insert in the zeocin-resistant yeast clones was validated by PCR screening using the primer pair pBlueYNTLfl_F and pBlueYNTRfl_R. Growth of the selected colonies was tested on the agarized media containing nitrate and nitrite (in the concentration range of 0.2–5 mM) to exclude the occurrence of other spontaneous mutations in the genome which may occur when yeast is transformed by the lithium method.

The mutant strain Δ*ynt1* (*ynt1: BleoR*(*ZeoR*), *leu2*, *ura3*) was additionally transformed with pCCUR2 and pCHLX integrative plasmids carrying the *URA* and *LEU* genes, respectively, to ensure the growth of the yeast strains without additional nitrogen sources in the selective media, namely, leucine and uracil, when performing complementation tests.

### 2.10. Cultivation of H. polymorpha and Transformants of Δynt1 Strain

Cells of *H. polymotpha* WT strain, mutant strain Δ*ynt1*, and transformants of the Δ*ynt1* strain were grown on a rich YPD medium (1% yeast extract, 2% peptone, 2% glucose) or a minimal synthetic SD medium (0.67% yeast nitrogen base without amino acids, 2% glucose) with (NH_4_)_2_SO_4_ addition (medium SD + AS) or KNO_3_ addition (medium SD + KNO_3_) as nitrogen sources. The KNO_3_ concentrations ranged from 0.2 to 5 mM. The media contained or did not include auxotrophic additions (0.02 mg/mg leucine, 0.04 mg/mL uracil). The yeast growth occurred at 37 °C for 2–3 days. All manipulations with *H. polymorpha* were performed according to the protocols generally accepted for the yeast [51]. Yeast transformants were selected on minimal selective media in the absence of leucine and/or uracil. Transformants that contained the insertion in the genome were validated by PCR with DL-1_Chr2_HpURA3_F or Hp_DL-1_Chr1_R primers for genomic DNA and standard M13_R or M13_F primers for pCCUR2 and pCHLX vectors.

### 2.11. Study of nitrate uptake by H. polymorpha cells expressing SaNPF6.3 and AtNPF6.3 Genes

Cells of *H. polymorpha* knockout mutant strains Δ*ynt1* expressing *SaNPF6.3* and *AtNPF6.3*, as well as WT cells, were grown to approximately 20 mg fresh weight/mL in a minimal SD medium (2% dextrose and 0.67% YNB containing sulphate ammonium) at 37 °C for 2 days. To induce the expression of nitrate transporter genes, cells were precipitated, washed with water, and cultured for 24 h (37 °C) in a minimal SD–AS medium (2% dextrose, 0.67% YNB without ammonium sulphate) supplemented with 20 mM NaNO_3_ as the only nitrogen source. After induction of the nitrate transporter genes, yeast cells were repeatedly washed with water and resuspended in a minimal SD–AS medium (2% dextrose; 0.176% YNB without sulphate ammonium) supplemented with 0.5 mM or 2 mM NaNO_3_. The yeast cultures were left to grow in these media for 18 h (37 °C); the absorption of nitrate by the cells was determined by measuring nitrate concentration decline in incubation media using an Elite-021 NO_3_^−^ selective electrode (Niko-Analit, Moscow, Russia).

### 2.12. Immunoblotting

For SDS-PAAG protein electrophoresis and the following immunoblot analysis, *H. polymorpha* WT cells and cells transformed with pCHLX-*GFP-SaNPF6.3* were grown in 10 mL of SD + AS medium (0.67% yeast nitrogen base without amino acids, 2% glucose; ammonium sulphate as a nitrogen source was a component of the yeast nitrogen base) for 24 hr. Then, the yeast cells were transferred to SD + KNO_3_ medium (0.17% yeast nitrogen base without amino acids and ammonium sulphate, 2% glucose + 10 mM KNO_3_ as a sole nitrogen source). After cultivation for 24 h in the NO_3_^−^-containing medium, leading to the induction of nitrate transporter genes, the cells were precipitated and lysed in 300 µL of A buffer (20 mM Tris-HCl, pH 8.0, 100 mM NaCl, 1% SDS, 1 mM PMSF) with 300 µL glass beads using Vortex. The lysate was centrifuged (13,000× *g*, 10 min, +4 °C) and the proteins from the supernatant were analyzed by SDS-PAAG electrophoresis and immunoblotting. The protein concentration in the supernatant was determined by the bicinchoninic acid method [58]. Electrophoresis was performed in 8% separating gel, loading 50 µg of protein per lane. The semi-dry transfer of the proteins from PAAG matrix to the nitrocellulose membrane (NC) (pore diameter of 0.45 µm; Schleicher & Schuell, Dassel, Germany) was carried out at 100 mA for 1 h using transfer device (Helicon, Moscow, Russia). For immunodetection of the recombinant protein GFP-SaNPF6.3, polyclonal anti-GFP antibodies (Evrogen, Moscow, Russia) were used as primary antibodies and horseradish peroxidase-conjugated (HRP) antibodies (Imtek Ltd., Moscow, Russia) as secondary ones. After treatment with a blocking solution (5% Carnation non-fat dry milk (Nestle, Vevey, Switzerland) in TBS with 0.1% Tween-20), the membrane was incubated with the primary antibodies (overnight at +4 °C) and then with the secondary antibodies (2 h). Primary antibodies were used at a 1:2000 dilution in TBS–Tw–BSA solution (1×TBS, 0.2% Tween 20, 1% BSA, 0.01% NaN_3_), and secondary antibodies were used at a 1:10,000 dilution in the TBS–Tw–DM solution (1×TBS, 0.2% Tween 20, 5% Carnation non-fat dry milk). After incubation with the antibodies, the membrane was washed with TBS–Tw solution (1×TBS, 0.1% Tween 20) and immunoreactive bands were visualized using an ECL kit (GE Healthcare, Piscataway, NJ, USA). A ChemiDoc XRS+ System gel documentation system (BioRad, Hercules, CA, USA) was used for visualization.

### 2.13. Determination of SaNPF6.3 Localization in Yeast Cells

The images of yeast cells transformed with pCHLX-*GFP-SaNPF6.3* were obtained with a laser scanning confocal microscope LSM-710-NLO (Carl Zeiss, Jena, Germany) equipped with a 63x oil immersion objective Plan-Apochromat, with numerical aperture of 1.4 and ZEN 2010 software 1.1.2.0 (Carl Zeiss, Jena, Germany). For analysis, the suspension of yeast cells was transferred to sterilized Petri dishes with a thin glass bottom. The fluorescence signals were detected in confocal channel mode with a confocal diaphragm of 46 μm in diameter, image size 1024 × 1024 pixels (132 nm/pixel), and a scanning rate of 1.27 μs/pixel (1.33 s/image). The GFP fluorescence was excited at λ_ex_ = 488 nm and visualized in the 490–555 nm range. The transmitted laser light was recorded with a separate T-PMT detector.

### 2.14. Quantitative Analysis of SaNPF6.3 Transcripts in S. altissima Organs

Quantitative analysis of *SaNPF6.3* transcripts was performed by qRT-PCR using a LightCycler^®^ 96 System (Roche Diagnostics Corporation, Indianapolis, IN, USA). The cDNA templates for the amplification of *SaNPF6.3* fragments were synthesized on total RNAs templates, isolated from different organs of *S. altissima* plants grown in the NS with various nitrate and NaCl concentrations or the plants subjected to NaCl shock. A ready-to-use reaction mixture with intercalating dye SYBR Green I (Evrogen, Moscow, Russia) was used. The *S. altissima* gene of elongation factor 1 alpha *SaeEF1alpha* (GenBank ID: MN076325.1) and protein phosphatase gene *SaPP2A* (GenBank ID:OP752355) were used as the reference genes. To amplify the *SaeEF1alpha* and *SaPP2A* fragments, SaeEF1alfa_F1 and SaeEF1alfa_R1, and SaPP2A_F1 and SaPP2A_R1 primer pairs, respectively, were used. The results are based on three replicates. The results obtained were processed by the LightCycler 96SW 1.1 software. Similar results were obtained with *SaeEF1alpha* and *SaPP2A,* hence, the data are presented only for the former gene. For the amplification of *SaNPF6.3* fragment, the primer pair SaNPF6.3_F1 and SaNPF6.3_R1 was used.

### 2.15. Determination of NO_3_^−^ and Cl^−^ Concentrations in Xylem Exudates

Xylem exudates were mineralized at 400 °C, the solid residues obtained were diluted with mQ water and the nitrate concentrations in the solutions were determined using an Elite-021 NO_3_^−^-selective electrode (Niko-Analit, Moscow, Russia). Cl^−^ was assayed by titration with Hg^2+^ using a Top Buret H digital burette (Eppendorf, Wesseling-Berzdorf, Germany).

### 2.16. Bioinformatic Analysis of Amino Acid Sequences

Multiple sequence alignment of amino acid sequences was performed by MAFFT software, version 7 (https://www.ebi.ac.uk/Tools/msa/mafft/, accessed on 4 July 2023) and visualized by Jalview software, version 2.11.2.7 (https://www.jalview.org/ (accessed on 4 July 2023)). A phylogenetic analysis of plant NPF family proteins was carried out by Molecular Evolutionary Genetic Analysis (MEGA) 11 software (version 11, https://www.megasoftware.net/, accessed on 4 July 2023), using the maximum likelihood method based on the Jones–Taylor–Thornton model [59] (1000 bootstrap replications were performed). Protein topology was predicted by DeepTMHMM software (version 1.0.24, https://dtu.biolib.com/DeepTMHMM, accessed on 26 September 2023). The 3D protein structure was predicted by SWISS-MODEL (https://swissmodel.expasy.org/interactive, accessed on 26 September 2023).

### 2.17. Statistics

Data processing (mean, standard errors) and the production of graphs were performed using Sigma Plot software (version 14.0). Statistical analysis of the data was made by one-way analysis of variance (ANOVA). Statistical calculations were carried out in the Microsoft Excel program (version 2019). Standard errors are given. Different letters indicate significant difference (*p*-value < 0.05).

## 3. Results

### 3.1. Full-Length Cloning and in Silico Analysis of the Protein SaNPF6.3

We had earlier obtained the partial coding sequence (CDS) of *SaNPF6.3* (GenBank ID: MK580125.1), the gene of a nitrate transporter from the halophyte *S. altissima*, based on the assumed similarity of *SaNPF6.3* to the gene of a nitrate transporter from the closely related species *S. fruticosa* [53]. Here, based on the partial CDS of *SaNPF6.3*, we determined the full-length cDNA of *SaNPF6.3* by a rapid amplification of 3′- and 5′- ends of cDNA. The entire coding sequence *of SaNPF6.3*, 1788-bp in size, was cloned into the yeast integrative vector pCHLX [54] and sequenced. The cloned *SaNPF6.3* sequence contained an open reading frame encoding a protein of 596 aa in size, with a calculated molecular mass of 66.0 kDa.

**Figure 1:**
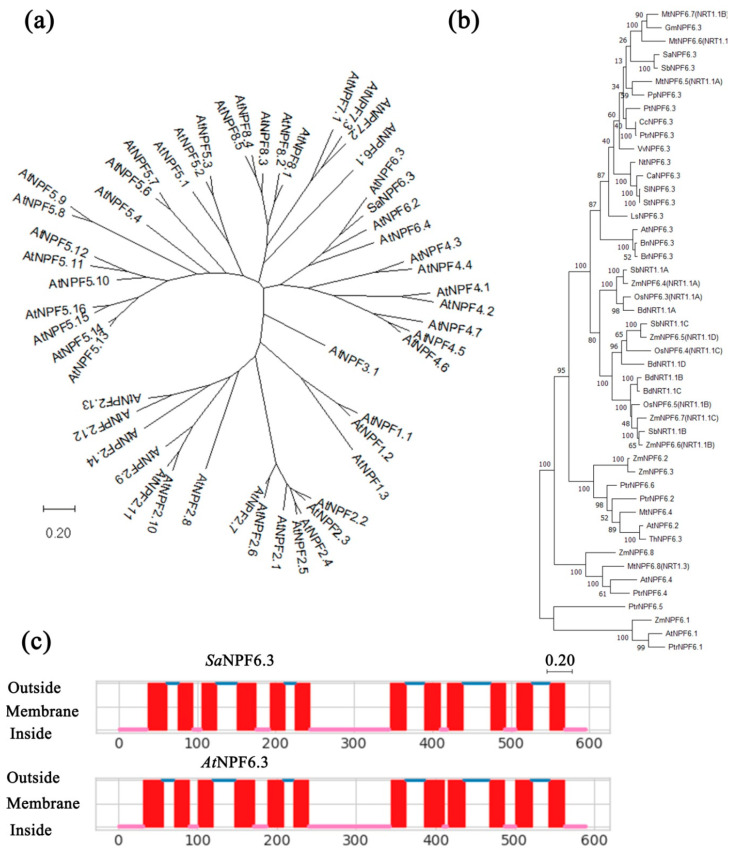
Phylogenetic analyses for plant NPF/NRT1/PTR family nitrate transporters (**a**,**b**) and SaNPF6.3 and AtNPF6.3 membrane topology (**c**). The phylogenetic trees (**a**,**b**) were built in the MEGA 11 using the maximum likelihood method based on the Jones–Taylor–Thornton model. The number of bootstrap replicates was 1000; the values of bootstrap support are indicated near the nodes. Scale: 0.2 (**a**) and 0.2 (**b**) substitutions per site. The protein sequences were taken from NCBI, UniProtKB and Salicornia DB. Names of plant objects and protein IDs are given in the Tables S2 and S3. The membrane topology of SaNPF6.3 (**c**) was predicted by DeepTMHMM software (version 1.0.24, https://dtu.biolib.com/DeepTMHMM; accessed on 26 September 2023). For comparison, AtNPF6.3 topology predicted by the same software is given.

Phylogenetic analysis showed that the protein SaNPF6.3 lies in a clade with the dual-affinity nitrate transporter AtNPF6.3 from *Arabidopsis* [7,14] (Figure 1a) and is most similar to the putative nitrate transporter SbNPF6.3 (Salicornia DB: Sbi_g26995.t1) from halophyte *Salicornia bigelovii,* compared to the other NPF6 transporters (Figure 1b).

According to the topology model predicted by the DeepTMHMM software, SaNPF6.3, like other characterized members of the NPF6.3 family, is an integral membrane protein forming 12 hydrophobic transmembrane α-helices (TMH) and a cytoplasmic hydrophilic loop of 101 aa between TMH6 and TMH7; both N- and C-ends are predicted to be cytosolic ones (Figure 1c), which is generally typical for the NPF/NRT1/PTR family members of nitrate transporters [16]. The predicted 3D structure of SaNPF6.3 (Figure S2) coincided with the topology model.

**Figure 2:**
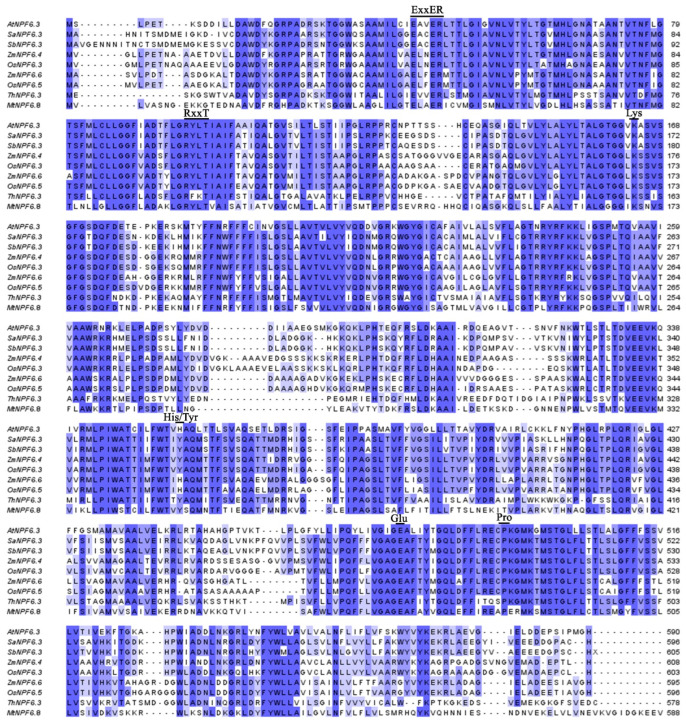
Multiple sequence alignment performed in MAFFT software for the proteins: SaNPF6.3 (GenBank ID: OQ330855), AtNPF6.3 (NRT1.1/CHL1) (GenBank ID: NP_563899.1), SbNPF6.3 (Salicornia DB: Sbi_g26995.t1), ZmNPF6.4 (GenBank ID: BT053880.1), ZmNPF6.6 (GenBank ID: XM_008660202.3), OsNPF6.3 (GenBank ID: XP_015650127.1), OsNPF6.5 (GenBank ID: XP_015614015.1), ThNPF6.3 (GenBank ID: BAJ33792.1) and MtNPF6.8 (GU966590.1). The key motifs (ExxER, RxxT) and amino acid residues (Lys, His/Tyr, Glu, Pro) are marked by lines above the sequences. The intensity of the staining of amino acid residues corresponds to the degree of their identity (Percentage Identity).

The SaNPF6.3 aa sequence was aligned with those of other plant NPF6 proteins (Figure 2 and Figure S1). Several key residues and motifs are conserved among SaNPF6.3 and other plant NPF6 proteins. At the N-terminus of SaNPF6.3, a conserved ExxER motif (EACER) was found at the position of 46–50 aa (Figure S2). This motif in SaNPF6.3 is likely a proton-binding domain, by analogy with AtNPF6.3, ZmNPF6.6 and ZmNPF6.4, as well as other NPF6 homologs [15,16]. Another conserved motif at the N-terminus of SaNPF6.3, RxxT (RYLT), with threonine residue at position 106, is probably the phosphorylation site (Figure S2). In SaNPF6.3, Thr106 is equivalent to Thr101 in AtNPF6.3 [11], Thr104 in ZmNPF6.6, Thr106 in ZmNPF6.4 [26] and Thr104 in MtNPF6.8 [27]. In *Arabidopsis*, the phosphorylation status of Thr101 was shown to control AtNPF6.3 affinity to nitrate [13,60]. The conserved histidine residue involved in nitrate binding in AtNPF6.3 (His356) [11] and present at equivalent positions in several other NPF6 homologues, namely, His362 in OsNPF6.5, His355 in BnNPF6.3 and His359 in SbNPF6.3 [16], is replaced by a tyrosine residue in SaNPF6.3 (Tyr358) (Figure 2 and Figure S2). Conserved residues of lysine (Lys168) and glutamate (Glu482) were also revealed in SaNPF6.3, of which the equivalents in AtNPF6.3 are Lys164 and Glu476 that form the ionic bond (salt bridge) required for maintenance of the protein structure in a functional state [15,16]. A disulfide bond between Cys130 and Cys137, stabilizing the extracellular loop between TMH3 and TMH4 in AtNPF6.3 [16], is apparently formed by Cys135 and Cys142 in SaNPF6.3 (Figure 2). In SaNPF6.3, a conserved proline residue (Pro498) is also present (Figure 2), the equivalent of which (Pro492) plays a key role in AtNPF6.3 signaling functions [18]. Similar conserved motifs and key amino acids were found in homologs of AtNPF6.3 in explored transcriptomes of numerous plant species including halophytes (Table S3).

### 3.2. Functional Complementation of the Yeast Mutant Δynt1 by SaNPF6.3 Expression in the Yeast Cells

**Figure 3:**
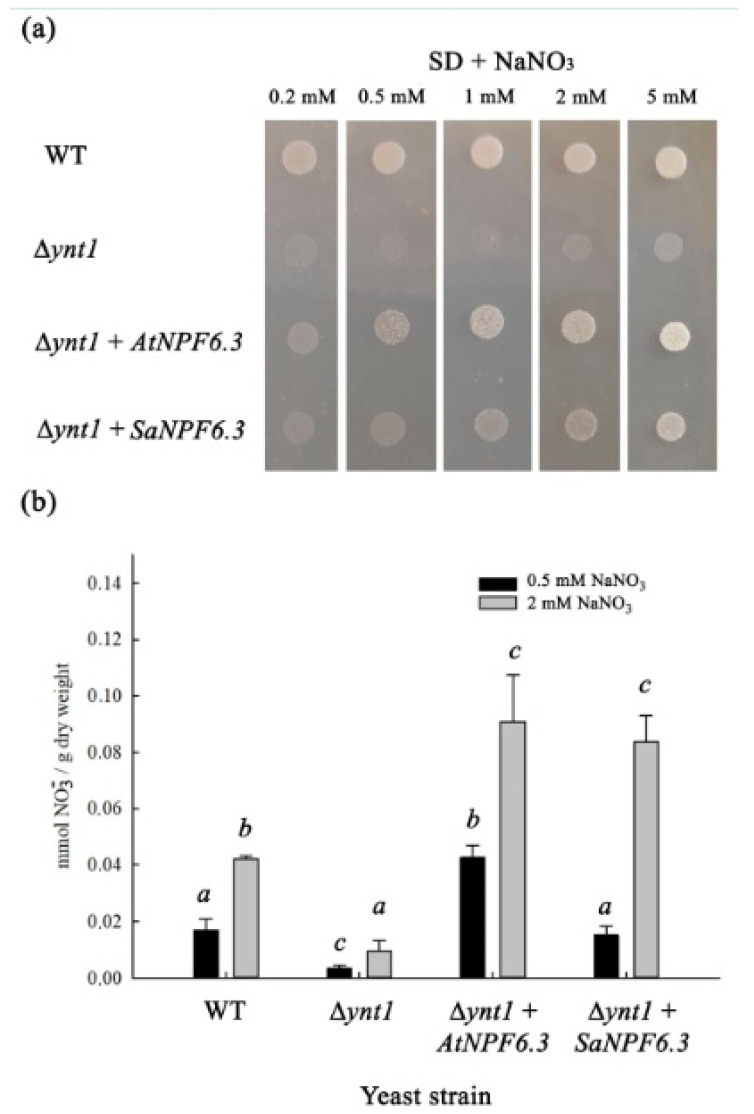
Complementation of growth and nitrate absorption defects of the yeast mutant strain Δ*ynt1* by expression of *SaNPF6.3* gene in the mutant cells. (**a**) Growth of the yeast mutant cells expressing *SaNPF6.3* on an agarized minimal SD media (0.17% yeast nitrogen base without amino acids and sulphate ammonium, 2% dextrose) containing NaNO_3_ in concentrations ranged from 0.2 to 5 mM. Approximately 10^5^ of the yeast cells were plated on the agarized selective media. The yeast colonies grew for 3 days at 37 °C. (**b**) Nitrate absorption from the liquid minimal SD media, containing NaNO_3_ in concentrations 0.5 or 2 mM, by the yeast mutant Δ*ynt1* transformed with the *SaNPF6.3* gene; the initial wet weight of the yeast suspension was 10 mg/mL. The yeast cells grew in the liquid media for 18 h at 37 °C. Controls: wild-type DL-1 strain and the mutant Δ*ynt1* strain, transformed with vectors pCHLX and pCCUR2, and the mutant Δ*ynt1* strain transformed with pCHLX*-AtNPF6.3* and pCCUR2. Different letters indicate significant difference (*p*-value < 0.05).

Most of the NPF6 members are thought to be nitrate transporters [61]. To demonstrate the nitrate transporting function of the SaNPF6.3 coding sequence, *SaNPF6.3* was expressed in the cells of the knockout mutant strain Δ*ynt1* of the methylotrophic yeast *H. polymorpha*. Growth of Δ*ynt1* strain was inhibited on a minimal SD medium containing NO_3_^−^ at concentrations ranging from 0.2 to 5 mM (SD + nitrate media) (Figure 3a). As a positive control for the complementation of the Δ*ynt1* mutation, heterologous expression of *AtNPF6.3* cloned from *A. thaliana* was used. Expression of *AtNPF6.3* in the mutant yeast cells, as in the earlier study [48], resulted in the recovery of the Δ*ynt1* colony growth on the (SD + nitrate) media. When Δ*ynt1* cells were transformed with *SaNPF6.3,* a recovery of the Δ*ynt1* growth on the (SD + nitrate) media also occurred (Figure 3a), indicating the involvement of SaNPF6.3 protein in NO_3_^−^ transport.

To validate a nitrate transporting function of SaNPF6.3, nitrate absorption by the cells of yeast transformants expressing *SaNPF6.3* was studied (Figure 3b). As a positive control, the yeast transformant expressing *AtNPF6.3* was used. In these experiments, the yeast cells were grown initially in the medium containing ammonium as the only nitrogen source, and then, nitrate transporting systems were induced by cell transfer to the nitrate medium. The WT yeast cells took up nitrate from the media containing this anion at concentrations of both 0.5 mM and 2 mM. The knockout mutation Δ*ynt1* resulted in a sharp decrease in nitrate uptake by the cells compared to WT at both nitrate concentrations, indicating the essential contribution of YNT1 in nitrate absorption by this organism. Expression of *SaNPF6.3*, like *AtNPF6.3*, in the mutant strain cells resulted in a recovery of nitrate uptake; the recovery occurred to an even higher level than in WT cells (Figure 3b). The results obtained indicate the ability of SaNPF6.3 to transport nitrate at nitrate concentrations in both high- and low-affinity ranges in the medium. Unfortunately, we were not able to assess the SaNPF6.3 chloride transport properties in the experiments with *H. polymorpha* mutant strain Δ*ynt1* expressing *SaNPF6.3*, presumably because of the endogenic Cl^−^-transporting activity of yeast cells (Figure S3).

We also examined the effects of substitutions of Thr106 by Ala disrupting phosphorylation and Thr106 by Asp mimicking constitutive phosphorylation on the growth of the yeast transformants as well as the substitution of Tyr358 by His, found in a number of NPF6.3 homologs. However, SaNPF6.3 with the above substitutions complemented the growth defect phenotype of the yeast mutant as effectively as the wild-type SaNPF6.3.

**Figure 4:**
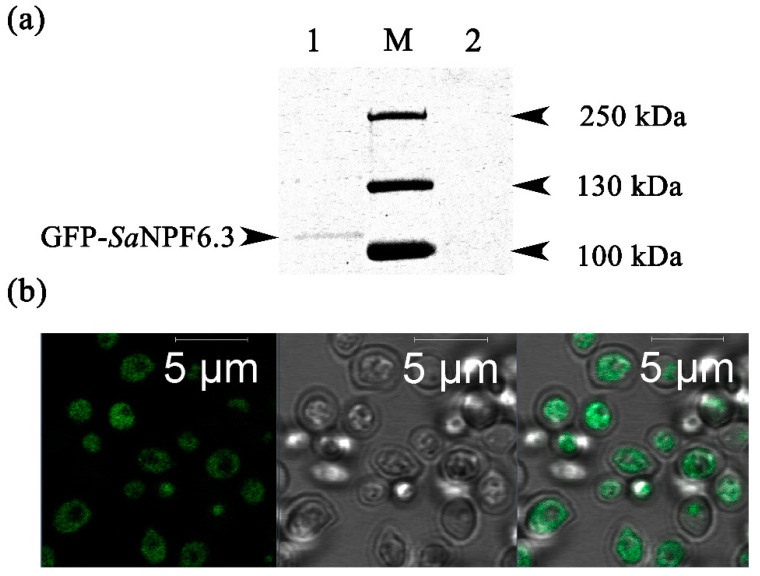
Evidence for expression of *GFP-SaNPF6.3* in the cells of the *H. polymorpha* mutant strain Δ*ynt1*. (**a**) Western blotting of cell lysates with a primary antibody to GFP. Lane 1—the mutant strain Δ*ynt1* expressing *GFP-SaNPF6.3*; lane 2—the mutant strain Δ*ynt1* (control); lane M—protein markers. (**b**) Confocal microscopy of *H. polymorpha* Δ*ynt1* cells expressing *GFP-SaNPF6.3*. Left—*GFP- SaNPF6.3* fluorescence; middle—bright field; right—merged image.

We investigated the localization of SaNPF6.3 in the cells of Δ*ynt1* transformants. For this purpose, the GFP coding sequence was fused to the 5′- end of the *SaNPF6.3*. and cells of the Δ*ynt1* mutant strain were transformed with the recombinant gene. At first, the presence of the GFP-SaNPF6.3 protein in cell lysates of the transformants obtained was determined by Western blotting with antibodies to GFP (Figure 4a). There was no band with a calculated molecular mass of 94.1 kDa for the GFP-SaNPF6.3 chimeric protein. However, a band of approximately 110 kDa was observed. The higher molecular mass of the observed band than the calculated molecular mass for GFP-SaNPF6.3 could be explained by an additional phosphorylation and glycosylation of the protein in the yeast heterologous system [62].

Using the approach based on the Western blot analysis, we selected colonies of the yeast transformants with the highest GFP-SaNPF6.3 expression. Confocal laser microscopy of the transformant cells revealed GFP fluorescence as clusters with a dot structure, localized in the cytoplasm and/or vacuoles. (Figure 4b). We did not observe significant GFP fluorescence in the plasma membranes of the transformants. Apparently, in the used heterologous expression system, just a small fraction of the fused GFP-SaNPF6.3 protein reaches the plasma membrane, which is insufficient to see intensive GFP fluorescence in the PM. Nevertheless, it seems that even a relatively small fraction of the transporter that was delivered to the PM provided complementation of the Δ*ynt1* growth-defect phenotype and observed nitrate uptake (Figure 3).

### 3.3. Quantitative Analysis of SaNPF6.3 Transcripts in S.altissima Organs

**Figure 5:**
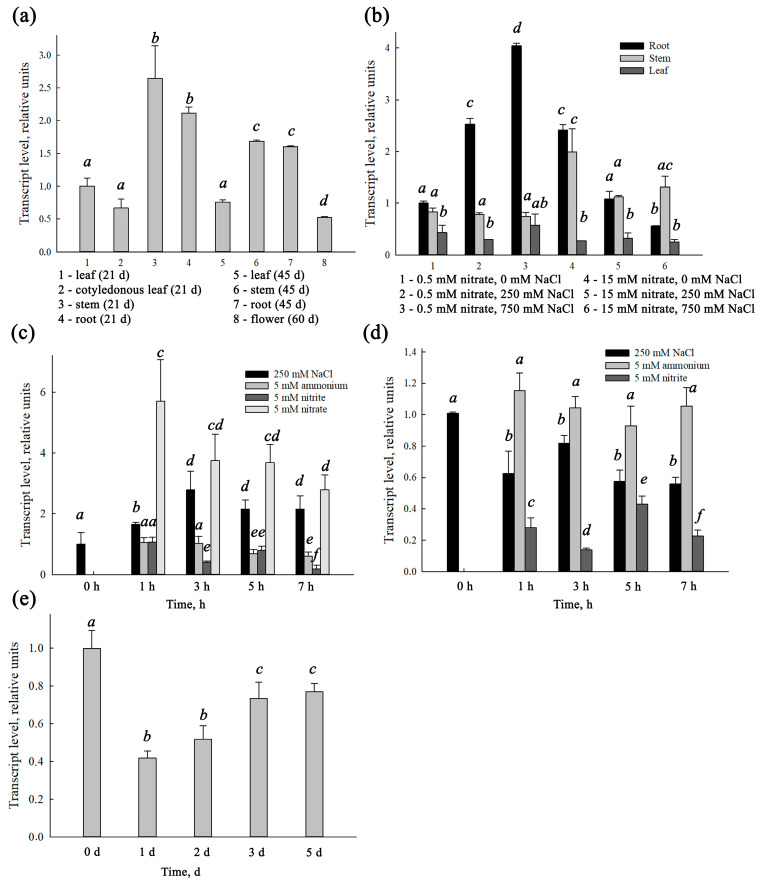
Relative abundance of *SaNPF6.3* transcripts in *S. altissima* plants. (**a**) *SaNPF6.3* expression in different organs and at different stages of the plant growth. (**b**) *SaNPF6.3* expression in roots, stems and leaves at different NaCl concentrations in NS, at the background of either low (0.5 mM) or high (15 mM) nitrate availability. (**c**) Dynamics of *SaNPF6.3* expression in roots of plants grown under conditions of low nitrate availability, following addition to the nutrient solution of 250 mM NaCl, 5 mM KNO_2_, 5 mM (NH_4_)_2_SO_4_) or adjustment of KNO_3_ concentration up to 5 mM. (**d**) Dynamics of *SaNPF6.3* expression in roots of plants grown under conditions of high nitrate availability, following addition to NS of 250 mM NaCl, 5 mM KNO_2_ or 5 mM (NH_4_)_2_SO_4_. (**e**) Dynamics of *SaNPF6.3* expression in roots of plants grown under conditions of high nitrate availability, following the transfer of the plants to the same medium but without nitrate. Data shown are mean ± SD from three independent experiments. Bars with different letters are significantly different at *p* < 0.05. The results were deduced from three biological replicates and each of them was performed in three analytical replicates.

We investigated expression of *SaNPF6.3* in organs of *S. altissima* at different stages of plant growth, at different nitrate concentrations in the NS and at different salinities (Figure 5). *SaNPF6.3* was expressed in all *S. altissima* organs tested: roots, stems, leaves and flowers. The expression levels were higher in the roots and stems than in the leaves and flowers (Figure 5a,b). *SaNPF6.3* was expressed both at a sufficient supply of nitrate to plants (15 mM NO_3_^−^) and under nitrate deficient conditions (0.5 mM NO_3_^−^) in the NS (Figure 5b). *SaNPF6.3* expression in the roots was significantly influenced by NaCl and depended on the NO_3_^−^ concentration in the nutrient solution. The *SaNPF6.3* transcript abundance in the roots increased at the low NO_3_^−^ concentration but decreased at the high concentration, when NaCl concentration increased from 0 mM to 750 mM NaCl (Figure 5b), mainly confirming the earlier results [53]. In the leaves, *SaNPF6.3* expression did not change significantly with variations of NaCl or NO_3_^−^ concentrations in the NS. In the stems, *SaNPF6.3* expression increased with increasing NO_3_^−^ concentration but was not influenced by NaCl concentration in the NS (Figure 5b).

We also examined the dynamics of *SaNPF6.3* expression changes in the roots of plants grown in the presence of either low (0.5 mM) or high (15 mM) NO_3_^−^ concentrations, following the additions of NaCl (250 mM), NaNO_2 (_5 mM) or (NH_4_)_2_SO_4_ (5 mM) to the NS (Figure 5c,d; the final concentrations are indicated). For plants grown at 0.5 mM NO_3_^−^ (Figure 5c), the concentration of KNO_3_ was also increased up to 5 mM. The expression of *SaNPF6.3* changed most significantly in the roots of plants grown in the NS with low NO_3_^−^ in response to an increase in NO_3_^−^ concentration (Figure 5c). Salt shock also led to increased *SaNPF6.3* expression, while nitrite inhibited it. In the roots of plants grown in the NS with 15 mM NO_3_^−^, the addition of nitrite and, to a lesser extent, NaCl shock, lowered *SaNPF6.3* transcript abundance. The transfer of plants grown in the NS containing 15 mM NO_3_^−^ to the NS without NO_3_^−^ led, in general, to decreasing *SaNPF6.3* expression in the roots (Figure 5e).

### 3.4. Determination of NO_3_^−^ and Cl^−^ Concentrations in Xylem Exudate

**Figure 6:**
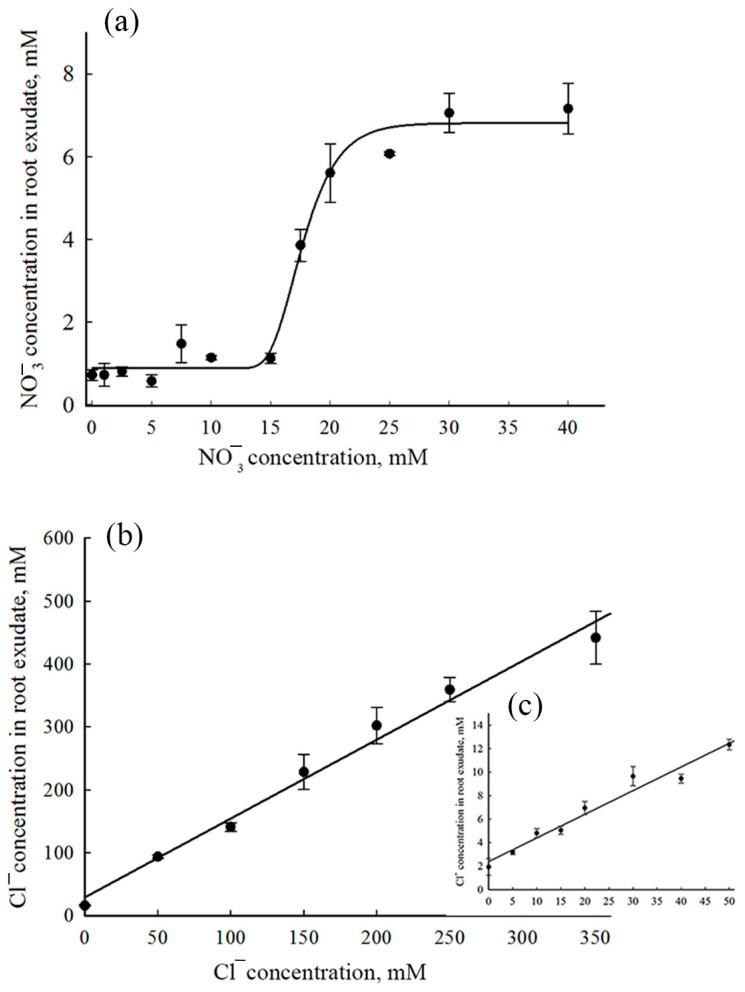
Concentrations of NO_3_^−^ and Cl^−^ in xylem exudate of *S. altissima* as functions of these anion concentrations in the nutrient solutions. (**a**) Dependence of NO_3_^−^ concentration in xylem exudate on NO_3_^−^ concentration in NS. (**b**) Dependence of Cl^−^ concentration in xylem exudate on Cl^−^ concentration in NS. (**c**) The same as (**b**), but the dependence is presented in a smaller range of the external concentrations. Cl^−^ concentrations are indicated in the medium along abscissa and in the xylem exudate along ordinate.

The low-affinity NPF/NRT1/PTR and high-affinity NRT2 transporter families, as well as the slow anion channel family SLAC/SLAH, play key roles in NO_3_^−^ and Cl^−^ uptake by the roots and the root-to-shoot translocation of these anions [10,63,64]. The NO_3_^−^ and Cl^−^ content in the xylem exudate is seemingly a result of the joint functioning of the proteins of these families. To gain an insight into whether identical mechanisms are involved in the transport of both NO_3_^−^ and Cl^−^ in *S. altissima* roots, we determined the NO_3_^−^ and Cl^−^ concentrations in xylem exudates as functions of concentrations of these anions in the NS. For this purpose, we removed the shoots of plants growing at different concentrations of NO_3_^−^ and Cl^−^ in the NS and measured concentrations of these anions in the exudates collected. The NO_3_^−^ concentration in the exudates held at a stable level of about 1 mM when the NO_3_^−^ concentration in the NS increased in the range of 0 mM to 15 mM but sharply increased up to 6.5–7.0 mM at external NO_3_^−^ concentrations of 15–20 mM and remained at this level at higher NO_3_^−^ concentrations in the NS (Figure 6a). The dependence of Cl^−^ concentrations in exudates on the Cl^−^ concentration in the NS was of another pattern. In xylem exudates excreted by *S. altissima* roots, Cl^−^ concentrations rose linearly as NaCl concentrations increased, and the linearity remained over a wide range of external NaCl concentrations, up to 350 mM (Figure 6b,c).

## 4. Discussion

From the euhalophyte *Suaeda altissima*, we cloned the coding sequence of the *SaNPF6.3* gene, homologous to the *AtNPF6.3*/*AtNRT1.1* encoding a dual-affinity nitrate transporter/transceptor in the model plant *A. thaliana*. The protein SaNPF6.3 belongs to the large NPF/NRT1/PTR family of transporters, including proteins involved in the transport of nitrate, nitrite, peptides, amino acids, glucosinolates, auxin, ABA and gibberellins [10,13,65]. Phylogenetic analysis showed evolutionary relations of SaNPF6.3 to NPF6.3 family proteins (Figure 1) and displayed a larger similarity of SaNPF6.3 to NPF6.3 members from other families of dicotyledonous plants rather than families of monocotyledons. A SaNPF6.3 topological model matched those of the NPF6.3 proteins from other plants, in particular AtNPF6.3 (Figure 2b). In silico analysis based on SaNPF6.3 multiple sequence alignment with homologous proteins from other plants (Figure 2) revealed that SaNPF6.3 has features inherent in the NRT1/NPF/PTR family of anion transporters.

**(1)** A conserved threonine residue, Thr106, in the motif RxxT (Figure 2), a putative phosphorylation site, is found at the N-terminus of SaNPF6.3 in TMH3 (Figure 1c). Similar threonine residues are present at equivalent positions in other NPF6.3 transporters. In AtNPF6.3, an equivalent Thr101 was shown to be responsible for switching between a high- and a low-affinity AtNPF6.3 mode [14,60]. The switching was accomplished through alterations in the Thr101 phosphorylation status which was dependent on nitrate availability in the growth medium. At low nitrate concentrations (<1 mM), Thr101 is phosphorylated by CBL-INTERACTING PROTEIN KINASE 23 (CIPK23) and AtNPF6.3 operates as a high-affinity transporter. In the range of millimolar nitrate concentrations, Thr101 is dephosphorylated and AtNRT1.1 operates as a low-affinity nitrate transporter [14,60]. However, the anion-transporting activities of *Zea mays* proteins, ZmNPF6.6 and ZmNPF6.4, unlike AtNPF6.3, appear unlikely to be regulated by the phosphorylation status of equivalent threonine residues [26]. In ZmNPF6.6, the point mutations Thr104Ala disrupting phosphorylation and Thr104Asp mimicking a phosphorylation event retained the high-affinity nitrate transport activity, although with a lower Km for nitrate. Thr106Ala and Thr106Asp substitutions in chloride-transporting ZmNPF6.4 eliminated the saturable curve in chloride uptake, with influx activity becoming linear as external chloride concentrations increased [26]. The authors suggested that the transport activity of ZmNPF6.6 and ZmNPF6.4 changed in a phosphorylation-independent but threonine-dependent manner. We made similar substitutions in the *S. altissima* protein: Thr106Ala and Thr106Asp. Growth of the mutant yeast strain Δ*ynt1* transformed with the modified and unmodified versions of *SaNPF6.3* on the minimal SR medium containing nitrate did not differ markedly (data not presented), testifying against the role of a Thr106 phosphorylation status in switching SaNPF6.3 affinity to nitrate.

**(2)** In TMH7 of SaNPF6.3, there is tyrosine residue Tyr358 (Figure 1c and Figure 2), the equivalent position of which is occupied by His in a number of NPF6.3 homologues including AtNPF6.3 [11], ZmNPF6.6 [26] and several others [16]. The mutation of His356Ala abolished both high- and low-affinity nitrate transporter activities in AtNPF6.3, suggesting that His356 is required for nitrate binding [15,16]. SaNPF6.3 is not the only NPF6.3 transporter containing Tyr at the equivalent positions (Figure 2, Table S3). Tyr was also found in dual-affinity nitrate transporters MtNPF6.8 (Tyr350) [27] and OsNPF6.3 (Tyr366) [25], as well as in the high-affinity chloride and low-affinity nitrate transporter ZmNPF6.4 (Tyr370) [26]. In a comparative study of anion transport by ZmNPF6.6 containing His362 and ZmNPF6.4 containing Tyr370, the authors found that in the absence of chloride, ZmNPF6.6 operates as a pH-dependent non-biphasic high-affinity nitrate-specific transporter, while ZmNPF6.4 acts as a low-affinity nitrate transporter. In the presence of chloride, ZmNPF6.6 switched to low-affinity, while ZmNPF6.4 to a high-affinity chloride transporter [26]. The authors hypothesized that ZmNPF6.4 *in planta* is likely to be a component of the root chloride uptake system. SaNPF6.3 bearing Tyr358 should presumably share more features with ZmNPF6.4 than with ZmNPF6.6 and function more as a transporter of chloride than of nitrate. However, SaNPF6.3 transported nitrate in a test with functional complementation of the yeast mutant Δ*ynt1*, lacking the only nitrate transporter (Figure 3a,b). **(3)** Analogs of the conserved residues (Lys165 and Glu476), forming ionic bonds to maintain the AtNPF6.3 in a functional state [15,16], were found in SaNPF6.3 (Lys168 and Glu482) (Figure 2). **(4)** In TMH1 of SaNPF6.3, there is an ExxER motif (EACER) conserved among NPF6.3 family members (Figure 2), an analog of which (EAVER) in AtNPF6.3 is involved in proton binding and the coupling of anion and proton transport [15,16]. **(5)** The presence of conserved proline residue between TMH10 and TMH11 was found to be a common property of all examined NPF6.3 homologs including AtNPF6.3 (Pro492) (Figure 2) [15,16]. This proline residue is essential for signaling functions of AtNPF6.3 [18]. We found an analog of this residue in SaNPF6.3 (Pro498) (Figure 2).

SaNPF6.3 is likely an ortholog of AtNPF6.3/NRT1.1, a dual-affinity nitrate transporter/transceptor from *A. thaliana*. The latter assumption is based on several points. **(I)** The *SaNPF6.3* expression occurred under a variety of environmental conditions similar to the expression of the *AtNPF6.3,* the gene encoding dual-affinity nitrate transporter AtNPF6.3. The expression of *SaNPF6.3* was observed under external nitrate concentrations corresponding to both high- and low-affinity modes of the protein operation, as well as in the presence of the reduced nitrogen compounds, ammonium and nitrite ions, at salinization of the medium and without it (Figure 5). The gene was transcribed in the root, stem, leaf and flower; the transcription occurred at different ontogenetic stages (Figure 5). Expression levels were higher in the roots and stems than in the other organs, indicating a particularly important role of SaNPF6.3 in nitrate uptake and transfer. **(II)** The recovery of *H. polymorpha* mutant strain Δ*ynt1* growth on the minimal SD medium, in the presence of nitrate, when *SaNPF6.3* was expressed in the yeast cells, demonstrated clearly the nitrate transport properties of the *S. altissima* protein (Figure 3a). It should be noted that the nitrate concentrations applied, from 0.2 to 5 mM NO_3_^−^, covered both high- and low-affinity ranges. The lack of full growth recovery of the Δ*ynt1* mutant transformed with *SaNPF6.3* may be due to a limited expression of this gene in the heterologous system used. Also, it is possible that only a small fraction of the synthesized SaNPF6.3 protein is delivered to the plasma membrane in the yeast cells, or a rapid degradation of the protein occurred. In line with this assumption, laser confocal microscopy demonstrated that in the mutant yeast cells expressing *GFP-SaNPF6.3,* the recombinant protein was localized in the cytoplasm and vacuole (Figure 4b). **(III)** Nitrate uptake by the mutant Δ*ynt1* cells expressing *SaNPF6.3* demonstrated directly a nitrate-transporting function of SaNPF6.3 (Figure 4b). The yeast transformants took up nitrate from the media containing this anion in concentrations of both 0.5 mM and 2.0 mM. **(IV)** Like *AtNPF6.3* [11,66], the induction of *SaNPF6.3* expression in response to an increase in nitrate concentration in the NS, when plants are grown at a low nitrate availability (Figure 5c), and the suppression of *SaNPF6.3* expression following the plants being transferred from sufficient nitrate supply to nitrate starvation (Figure 5e) support also the participation of SaNPF6.3 in nitrate uptake. The decreased expression of *SaNPF6.3* in response to a nitrite addition at low nitrate availability (Figure 4c) may be the result of an increased fraction of reduced nitrogen compounds in the cells during nitrate assimilation, which inhibits *SaNPF6.3* expression by the feedback.

It is possible that SaNPF6.3 mediates a low-affinity transport of chloride ions in addition to the dual-affinity transport of nitrate. The involvement of SaNPF6.3 in a low-affinity chloride transport is supported by increasing expression of the transporter gene in the response to NaCl addition to the NS. The stimulation of *SaNPF6.3* expression by NaCl in the presence of 0.5 mM nitrate observed at both the long-term salinity and under the salt shock conditions suggests the involvement of the transporter in the transport of chloride. *S. altissima,* like other salt-accumulating halophytes, absorbs chloride in large quantities as a counter-ion for Na^+^ passively entering the root cells. Further root-to-shoot translocation of Cl^−^ and Na^+^, which play a role of ‘cheap’ osmotic compounds, contributes to maintaining the water potential gradient in the soil-root-shoot system [43,67,68]. Another possible reason for the stimulation of *SaNPF6.3* expression in response to increasing NaCl concentration in the NS at low nitrate availability is a greater requirement for nitrate uptake under conditions of its competition with the chloride ion.

The decline in the expression of the transporter with increasing external NaCl concentration in the high external nitrate concentration (15 mM) may be a consequence of excessive amounts of the anions in the medium, both to meet the nitrogen requirements of plants and to maintain osmotic pressure in the cells. With the external NaCl concentration increasing, often accompanied by PM depolarization [69], the transition point from active to passive chloride uptake can be attained [70], decreasing the requirements of root cells in this transporter.

By collecting xylem exudate, we also examined the ability of *S. altissima* roots to deliver nitrate and chloride to the above-ground organs (Figure 6). The anion transfer to the shoots is based on the functions of the proteins responsible for absorption and root-to-shoot translocation of nitrate and chloride. The detached intact roots immersed in the nutrient solution represent a complex model system unable to demonstrate the function of individual transport proteins, such as SaNPF6.3. However, this system could be useful in ascertaining the physiological role of the protein operating in the whole organ, together with other proteins. As the ionic composition of the *S. altissima* root exudate showed (Figure 6), roots were able to ensure the delivery of both nitrate and chloride to the shoots. Chloride is required for the euhalophyte in a large quantity for the maintaining of cell turgor and the water potential gradient in the system of the whole plant [43]. The nitrate transporting activity of SaNPF6.3 suggests the SaNPF6.3 involvement in nitrate absorption by *S. altissima* roots. Two discrete levels observed for the NO_3_^−^ concentration in the exudate (Figure 6a) may be attributed to high- and low-affinity modes of SaNPF6.3. However, another possibility is that another protein belonging to the NPF/NRT1 or NRT2 family is involved in nitrate absorption along with SaNPF6.3, or two different slow anion channels are activated for xylem loading as nitrate concentration increases. The ability to transfer chloride, along with nitrate, was demonstrated for two NPF homologues from *Z. mays*, Zm-NPF6.6 and Zm-NPF6.4 [26]. Given that in each of the dicotyledons studied, unlike in monocotyledons, only one AtNPF6.3 homolog has been found [24], one would expect SaNPF6.3 to have properties that provide an ability for high-affinity nitrate transport in combination with low-affinity chloride transport.

## Data Availability

All data included in this study are available upon request by contact with the corresponding authors. The seeds of *Suaeda altissima* are available from the authors on request. The cloned *SaNPF6.3* cDNA was deposited in GenBank (acc. no. OQ330855).

## Supplementary Materials

The supporting information can be downloaded at: https://www.mdpi.com/article/10.3390/membranes13100845/s1, Figure S1. Alignment of the proteins from *Suaeda*, SaNRT1.1, SfNRT1.1 and SgNRT1.1.; Figure S2. SaNPF6.3 model predicted by Swiss-Model software; Figure S3. Chloride absorption from the liquid minimal SD media containing NaCl (3 or 10 mM) and NaNO_3_ (1 mM) by the yeast strains; Table S1. List of the primers used in the research; Table S2. List of the *A. thaliana NPF* genes and the corresponding proteins; Table S3. List of several gene sequences homologous to sequence *AtNPF6.3*.

## Abbreviations

CDS—coding sequence; CIPK 23—CBL-Interacting Protein Kinase 23; HATS—High-Affinity Transport System; LATS—Low-Affinity Transport System; NPF—Nitrate transporter 1/Peptide transporter Family; NRT—Nitrate Transporter; NS—Nutrient Solution; ORF—Open Reading Frame; PTR—Peptide Transporter; TMH—Transmembrane Helice; YNT1—Yeast Nitrate Transporter 1
