# Supplementary material for: Molecular Cloning, Expression and Transport Activity of SaNPF6.3/SaNRT1.1, a Novel Protein of the Low-Affinity Nitrate Transporter Family from the Euhalophyte Suaeda altissima (L.) Pall
Source: Membranes (Basel). 2023 Oct 22;13(10):845. doi: 10.3390/membranes13100845 (PMC10608580; doi:10.3390/membranes13100845)
Supplement: Supplementary file 1 [file membranes-13-00845-s001.zip › membranes-2564299-supplementary.pdf]

Supplementary materials

```
SaNRT1.1    1 MAHNITSMDMEIGKDIVCDAWDYKGRPAARSKTGGWQSAAMILGGEACERLTTLGIAVNLVTYLTGVMHLGNAASANTVTNFMGT  85
SfNRT1.1    1 MAHNITSMDMEIGKDTVCDAWDYKGRPADRSKTGGWQSAAMILGGEACERLTTLGIAVNLVTYLTGVMHLGNAASANTVTNFMGT  85
SgNRT1.1    1 MAHNITSMDMEIGKDTVCDAWDYKGRPADRSKTGGWKSAAMILGGEACERLTTLGIAVNLVTYLTGVMHLGNAASANTVTNFMGT  85

SaNRT1.1   86 SFMLCLLGGFIADTFLGRYLTIAIFATIQALGVTVLTISTIIPSLRPPKCEEGSDSCIPASDTQLGVLYLALYLTALGTGGVKAS 170
SfNRT1.1   86 SFMLCLLGGFIADTFLGRYLTIAIFATIQALGVTVLTISTIIPSLRPPKCEEGSDSCIPASDTQLGVLYLALYLTALGTGGVKAS 170
SgNRT1.1   86 SFMLCLLGGFIADTFLGRYLTIAIFATIQALGVTVLTISTIIPSLRPPKCVEGSDSCIPASDTQLGVLYLALYLTALGTGGVKAS 170

SaNRT1.1  171 VSGFGTDQFDESNKDEKLHMIKFFNWFFFFISLGSLAAVTILVYIQDNMGRQWGYGICACAIMLALVIFLCGTRRYRFKKLVGSP 255
SfNRT1.1  171 VSGFGTDQFDESNKEEKLHMIKFFNWFFFFISLGSLAAVTILVYIQDNMGRQWGYGICACAIMLALVIFLCGTRRYRFKKLVGSP 255
SgNRT1.1  171 VSGFGTDQFDESNKEEKLHMIKFFNWFFFFISLGSLAAVTILVYIQDNMGRQWGYGICACAIMLALVIFLCGTRRYRFKKLVGSP 255

SaNRT1.1  256 LTQIAAVFVAAWRKRHMELPSDSSLLFNIDDLADGGKHKKQKLPHSKQYRFLDRAAIKDPQMPSVVTKVNIWYLPTSTDVEEVKL 340
SfNRT1.1  256 LTQIAAVFVAAWRKRHMELPSDSSLLFNIDDLADGGKHKKQKLPHSKQYRFLDRAAIKDPQMPSVVTKVNIWYLPTSTDVEEVKL 340
SgNRT1.1  256 LTQIAAVFVAAWRKRHMELPSDSSLLFNIDDLADGGKHKKQKLPHSKQYRFLDRAAIKDPQMPSVVTKVNIWYLPTSTDVEEVKL 340

SaNRT1.1  341 VLRMLPIWATTIIFWTIYAQMSTFSVSQATTMDRHIGSSFRIPAASLTVFFVGSILITVPIYDRVVVPIASKLLHNPQGLTPLQR 425
SfNRT1.1  341 VLRMLPIWATTIIFWTIYAQMSTFSVSQATTMDRHIGSSFRIPAASLTVFFVGSILITVPIYDRVVVPIASRLLHNPQGLTPLQR 425
SgNRT1.1  341 VLRMLPIWATTIIFWTIYAQMSTFSVSQATTMDRSIGSSFQIPAASLTVFFVGSILITVPIYDRVVVPIASKILHNPQGLTPLQR 425

SaNRT1.1  426 IAVGLVFSIISMVSAALVEIRRLKVAQDAGLVNKPFQVVPLSVFWLVPQFFFVGAGEAFTYIGQLDFFLRECPKGMKTMSTGLFL 510
SfNRT1.1  426 IAVGLIFSIISMVSAALVEIRRLKVAQDAGLVNKPFQVVPLSVFWLVPQFFFVGAGEAFTYIGQLDFFLRECPKGMKTMSTGLFL 510
SgNRT1.1  426 IAVGLVFSIISMVSAALVEIRRLKVAQEAGLVNKPFQVVPLSVFWLVPQFFFVGAGEAFTYIGQLDFFLRECPKGMKTMSTGLFL 510

SaNRT1.1  511 TTLSLGFFVSSVLVSAVHKITGDKHPWIADNLNRGRLDYFYWLLAGLSVLNFLVYLLFAKWYVYKEKRLAEEGYIVEEEDDGPAC 595
SfNRT1.1  511 TTLSLGFFVSSVLVSAVHKITGDKHPWIADNLNRGRLDYFYWLLAGLSVLNFLVYLLFAKWYVYKEKRLADEGYIVEEEDDGPAC 595
SgNRT1.1  511 TTLSLGFFVSSVLVSAVHKITGDKHPWIADNLNRGRLDYFYWLLAGLSVLNFLVYLLFAKWYVYKEKRLAEEGYIVEEEDDGPAC 595

SaNRT1.1  596 H 596
SfNRT1.1  596 H 596
SgNRT1.1  596 H 596
```

Figure S1. Alignment of the proteins from *Suaeda*, SaNRT1.1, SfNRT1.1 and SgNRT1.1. The protein sequences of SfNRT1.1 and SgNRT1.1 were obtained by translation of the contigs in the *S. fruticosa* and *S. glauca* transcriptomes.

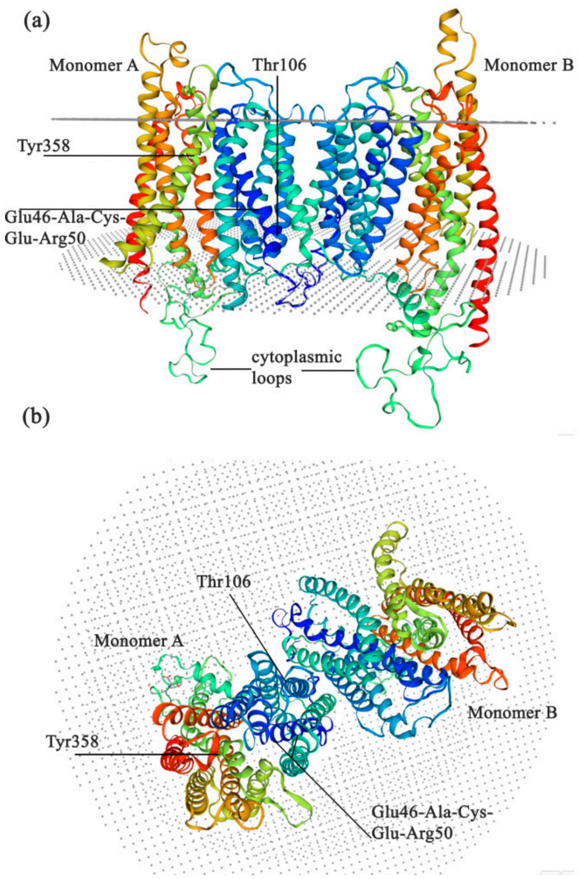

Figure S2. SaNPF6.3 model predicted by Swiss-Model software (https://swissmodel.expasy.org/ (accessed on 20 September 2023)). The key motifs (ExxER, EACER), amino acid residues (Thr106, Tyr358) and cytoplasmic loops are marked; (a) side view and (b) top view.

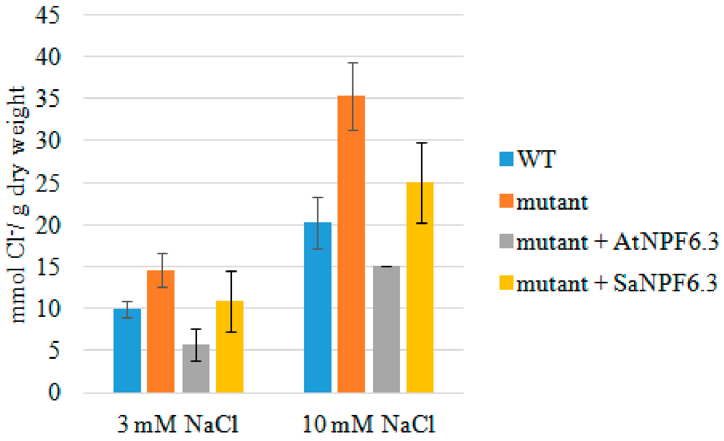

Figure S3. Chloride absorption from the liquid minimal SD media containing NaCl (3 or 10 mM) and $NaNO_3$ (1 mM) by the yeast strains; the initial wet weight of the yeast suspension was 10 mg/ml. Controls: wild type DL-1 strain and the mutant Δ*ynt1* strain, transformed with vectors pCHLX and pCCUR2, and the mutant Δ*ynt1* strain transformed with pCHLX-*AtNPF6.3* and pCCUR2. The yeast cells grew in the liquid media for 18 hours at 37°C; absorption of chloride by the cells was determined by measuring chloride concentration decline in incubation media using Cl^--selective electrode (Niko-Analit, Moscow, Russia).

Table S1. List of the primers used in the research.

Primer name	Nucleotide sequences (5´-3´)	Primer designation
SaNPF6.3_f	GACAGACCAATTCGACGAGA	Amplification of the cDNA central part of the SaNPF6.3 gene; Amplification of 5'- and 3'-fragments of SaNPF6.3 cDNA (1st round of PCR)
SaNPF6.3_r	CTAATAAGTAGTGTACGGTTGTCA	
SaNPF6.3_F1	CCTAGTCGAAATCAGGCG	Amplification of 5'- and 3'-fragments of SaNPF6.3 cDNA (2nd round of PCR);
SaNPF6.3_5′RACE_R1	GATGTTGGCAAGTACCAGATGTTG	
SaNPF6.3_R1	CTCCCACGAAGAAGAATTGAG	RT-qPCR with SaNPF6.3_F1 primer
ZeoYNTRfl_F	GGCTTTAATTTGCAATCGGCCGAACACGTGGTCAAGATCTGTTTTTCCC	Amplification of the right flanking sequence of the YNT1 locus to
pBlueYNTRfl_R	GGCTTTAATTTGCAATCGGCCGAACACGTGGTCAAGATCTGTTTTTCCC	

		obtain a deletion mutant Δ*ynt1*
pBlueYNTLfl_F	CTGCAGGAATTCGATCGGAGGTTCATGGATCAGGC	Amplification of the left flanking sequence of the YNT1 locus to obtain a deletion mutant Δ*ynt1*
YNTLflZeo_R	TGGTGTGTGGGGCGGCCGAACAAGAAGAGGCGGAAAAGCAAACC	
ZeoCas1	GTTCGGCCGCCCCACACACCATAGCTTCAAA	Zeocin resistance gene amplification
ZeoCas2	GTTCGGCCGATTGCAAATTAAAGCCTTCGAG	
SaNPF6.3_Gib_F	ATGGCTCATAATATTACTAGTATGGATATGGAAATTGG	SaNPF6.3 full-length coding sequence amplification from cDNA
SaNPF6.3_Gib_R	TTAGTGACATGCTGGCCCGT	
AtNPF6.3_Gib_F	ATGTCTCTTCCTGAAACTAAATCTGATG	AtNPF6.3 full-length coding sequence amplification from cDNA
AtNPF6.3_Gib_R	TCAATGACCCATTGGAATACTCGGC	
pYNR1_F	GGTCGACGGTATCGATAAGCTTATGGGGCTCCATATATCGTATGAC	amplification of the YNR1 nitrate reductase promoter
pYNR1_R	CCATACTAGTAATATTATGAGCCATTACAACTATCCAAAGTTCGTGAGGA	
tYNR1_F	CGGGCCAGCATGTCACTAACCTAAGCGAAATCGAAATCAAAACT	amplification of the nitrate reductase terminator
tYNR1_R	CCCGGGCTGCAGGAATTAATTCAGTATTTCAGAATCATGACCCC	

		YNR1
SaNPF6.3_1074_F	GCACAAATGAGTACTTTCTCCGTG	Introduction of Tyr358His amino acid substitution
SaNPF6.3_Tyr358His_R	ATGGATTGTCCAGAAAATAATAGTGGTG	
SaNPF6.3_318_F	ATTGCTATCTTTGCAACAATCCAAGC	Introduction of the amino acid substitution Thr106Asp or Thr106Ala
SaNPF6.3_Thr106Asp_R	ATCTAGATACCTTCCAAGGAAGGTGTCAG	
SaNPF6.3_Thr106Ala_R	AGCTAGATACCTTCCAAGGAAGGTGTC	
pYNR1_GFP_F	CACGAACTTTGGATAGTTGTAATGAGCAAGGGCGAGGAACTG	mGFP amplification
TEV_GFP_R	TGAAAATATAAATTTTCCCCGGGCTTGTACAGCTCGTCCATGC	
TEV_SaNPF6.3_F	CCCGGGGAAAATTTATATTTTCAAGGTCAATTTATGGCTCATAATATTACTAGTATGGATAT	SaNPF6.3 full-length coding sequence amplification from pCHLX-GFP-SaNPF6.3 cDNA
SaNPF6.3_Gib_R	TTAGTGACATGCTGGCCCGT	
pYNR1_R3	TACAACTATCCAAAGTTCGTGAGGA	Amplification of a linear form of pCHLX-pYNR1-tYNR1
DL-1_Chr2_HpURA3_F	GAGGTGAAGTCGTTGACGCA	PCR screening
Hp_DL-1_Chr1_R	TTGGTACCACCGCACTTCTC	PCR screening
AtNPF6.3_388R	ATCTTGGTGGTCGAAGTCCC	sequencing
AtNPF6.3_934F	ATCAGGAAGCGGGAGTTACC	
AtNPF6.3_1106R	GTCTCGGATTGTGCGACTGA	
pYNR1_R2	CATCAGATTTAGTTTCAGGAAGAGACATTACAACTATCCAAAGTTCGTGAGGA	Amplification of

tYNR1_F2	GCCGAGTATTCCAATGGGTCATTGACCTAAGCGAAATCGAAATCAAAACT	the nitrate reductase promoter and terminator
SaNPF6.3_1632R	GCGGTTCAAGTTGTCAGCAA	sequencing
SaNPF6.3_391R	GCAGGCTCGGGATTATGGTAG	
SaeEF1alfa_F1	TGAGATGTGTGGCAATCC	RT-qPCR
SaeEF1alfa_R1	GTTGCTTCTGACTCCAAGAAT	
SaPP2A_RT_F1	GAGCTTTTTCGTATCGGCGG	
SaPP2A_RT_R1	CCAGCAGCGTAACAGTCTCT	

Table S2. List of the *A. thaliana NPF* genes and the corresponding proteins.

Gene	Araport ID (TAIR)	UniProtKB ID
AtNPF1.1	AT3G16180	Q8LPL2
AtNPF1.2	AT1G52190	Q9M817
AtNPF1.3	AT5G11570	Q9LYD5
AtNPF2.1	AT3G45720	Q9M171
AtNPF2.2	AT3G45690	Q9M174
AtNPF2.3	AT3G45680	Q9M175
AtNPF2.4	AT3G45700	Q9M173
AtNPF2.5	AT3G45710	Q9M172
AtNPF2.6	AT3G45660	Q9M1E1
AtNPF2.7	AT3G45650	Q9M1E2
AtNPF2.8	AT5G28470	Q3E8X3
AtNPF2.9	AT1G18880	Q9MV7
AtNPF2.10	AT3G47960	Q944G5
AtNPF2.11	AT5G62680	Q9LV10
AtNPF2.12	AT1G27080	Q9LFX9
AtNPF2.13	AT1G69870	Q8RX77
AtNPF2.14	AT1G69860	Q9CAR9
AtNPF3.1	AT1G68570	Q9SX20
AtNPF4.1	AT3G25260	Q9LSF0
AtNPF4.2	AT3G25280	Q9LSE8
AtNPF4.3	AT1G59740	Q93VV5
AtNPF4.4	AT1G33440	Q56XQ6
AtNPF4.5	AT1G27040	Q8VYE4
AtNPF4.6	AT1G69850	Q8H157
AtNPF4.7	AT5G62730	Q9FM20
AtNPF5.1	AT2G40460	Q8VZR7
AtNPF5.2	AT5G46050	Q9FNL7
AtNPF5.3	AT5G46040	Q9FNL8
AtNPF5.4	AT3G54450	Q9M1I2
AtNPF5.6	AT2G37900	P0CI03
AtNPF5.7	AT3G53960	Q9M331
AtNPF5.8	AT5G14940	Q9LFR1
AtNPF5.9	AT3G01350	Q9SRI2
AtNPF5.10	AT1G22540	Q0WP01
AtNPF5.11	AT1G72130	Q8RX67
AtNPF5.12	AT1G72140	Q9C7U1
AtNPF5.13	AT1G72125	Q0WSZ6
AtNPF5.14	AT1G72120	Q8VZE2
AtNPF5.15	AT1G22570	Q9SK99
AtNPF5.16	AT1G22550	Q9SK96
AtNPF6.1	AT5G13400	Q9LYR6
AtNPF6.2	AT2G26690	Q9SZY4
AtNPF6.3	AT1G12110	Q05085
AtNPF6.4	AT3G21670	Q9LVE0
AtNPF7.1	AT5G19640	Q3E9B5
AtNPF7.2	AT4G21680	Q8GXN2
AtNPF7.3	AT1G32450	Q9LQL2
AtNPF8.1	AT3G54140	Q9M390
AtNPF8.2	AT5G01180	Q9LFB8
AtNPF8.3	AT2G02040	P46032
AtNPF8.4	AT2G02020	Q84WG0
AtNPF8.5	AT1G62200	Q93Z20

Table S3. List of several gene sequences homologous to sequence *AtNPF6.3*, key motifs and amino acid residues of the corresponding proteins.

Species	Protein name	Glu-x-x-Glu-Arg motif (ExxER)	Thr(T)	Lys (K)	His(H)/ Tyr(Y)	Glu (E)	Pro (P)	Gene ID (GenBank)
Arabidopsis thaliana	AtNPF6.3	41–45 Glu-Ala-Val-Glu-Arg (EAVER)	101	164	His 356	476	492	NP_563899.1
Suaeda altissima	SaNPF6.3	46–50 Glu-Ala-Cys-Glu-Arg (EACER)	106	168	Tyr 358	482	498	OQ330855
Thellungiella halophila	ThNPF6.3	38–42 Glu-Val-Val-Glu-Arg (EVVER)	98	159	Tyr 346	463	479	BAJ33792.1
Salicornia bigelovii	SbNPF6.3	54–58 Glu-Ala-Cys-Glu-Arg (EACER)	114	176	Tyr 366	490	506	Sbi_g26995.t1*
Medicago truncatula	MtNPF6.3	41–45 Glu-Ala-Cys-Glu-Arg (EACER)	101	164	Tyr 356	480	496	XP_003608752.1
	MtNPF6.8 (NRT1.3)	44–48 Glu-Leu-Ala-Glu-Arg (ELAER)	104	169	Tyr 350	465	481	GU966590.1
Zea mays	ZmNPF6.4 (NRT1.1A)	46–50 Glu-Leu-Asn-Glu-Arg (ELNER)	106	172	Tyr 370	493	509	BT053880.1
	ZmNPF6.6 (NRT1.1B)	44–48 Glu-Leu-Phe-Glu-Arg (ELFER)	104	169	His 362	479	495	XM_008660202.3
	ZmNRT1.1C	44–48 Glu-Leu-Phe-Glu-Arg (ELFER)	104	166	His 356	472	488	XM_008660209.3
	ZmNRT1.1D	49–53 Glu-Leu-Asn-Glu-Arg (ELNER)	109	176	Tyr 368	484	500	XM_008651564.2
Oryza sativa	OsNPF6.3(NRT1.1A)	47–51 Glu-Leu-Asn-Glu-Arg (ELNER)	107	169	Tyr 366	488	504	XP_015650127.1
	OsNPF6.5(NRT1.1B)	44–48 Glu-Leu-Phe-Glu-Arg (ELFER)	104	169	His 362	479	495	XP_015614015.1
	OsNPF6.4(NRT1.1C)	58–62 Glu-Leu-Asn-Glu-Arg	118	180	Tyr 369	482	498	XP_015632236.1

		(ELNER)						
Sorghum bicolor	SbNRT1.1A	46–50 Glu-Leu-Asn-Glu-Arg (ELNER)	106	170	Tyr 364	486	502	XM_002443834.2
	SbNRT1.1B	44–48 Glu-Leu-Phe-Glu-Arg (ELFER)	104	169	His 359	478	494	XM_002464914.2
	SbNRT1.1C	53–57 Glu-Leu-Asn-Glu-Arg (ELNER)	113	177	Tyr 374	490	506	XM_002468647.2
Populus trichocarpa (black cottonwood)	PtNPF6.3	40–44 Glu-Ala-Met-Glu-Arg (EAMER)	100	161	Tyr 347	471	487	XM_002303476.4
Brachypodium distachyon (purple false brome)	BdNRT1.1A	44–48 Glu-Leu-Asn-Glu-Arg (ELNER)	104	168	Tyr 363	484	500	XP_003573480.1
	BdNRT1.1B	48–52 Glu-Leu-Phe-Glu-Arg (ELFER)	108	173	His 365	481	497	XP_003574312.1
	BdNRT1.1C	52–56 Glu-Leu-Phe-Glu-Arg (ELFER)	112	177	His 367	483	499	XP_003572060.1
	BdNRT1.1D	64–68 Glu-Leu-Asn-Glu-Arg (ELNER)	124	189	Tyr 381	494	510	XP_003562233.3
Brassica napus (rapeseed)	BnNPF6.3	40–44 Glu-Ala-Val-Glu-Arg (EAVER)	100	163	His 355	475	491	XP_013698339.2
Brassica rapa (Chinese cabbage)	BrNPF6.3	40–44 Glu-Ala-Val-Glu-Arg (EAVER)	100	163	His 355	475	491	XP_009110669.1
Capsicum annuum (pepper)	CaNPF6.3	42–46 Glu-Ala-Val-Glu-Arg (EAVER)	102	164	Tyr 354	478	494	KAF3635032.1
Citrus	CcNPF6.3	41–45 Glu-Ala-Cys-Glu-Arg	101	164	Tyr 356	479	495	XP_006440344.1

clementina		(EACER)						
Glycine max (soybean)	GmNPF6.3	41–45 Glu-Val-Met-Glu-Arg (EVMER)	101	164	His 347	471	487	XM_003517379.5
Lactuca sativa (lettuce)	LsNPF6.3	41–45 Glu-Ala-Cys-Glu-Arg (EACER)	101	164	Tyr 359	483	499	XP_023738876.1
Nicotiana tabacum (tobacco)	NtNPF6.3	42–46 Glu-Ala-Val-Glu-Arg (EAVER)	102	165	Tyr 359	482	498	XP_016452049.1
Prunus persica (peach)	PpNPF6.3	44–48 Glu-Ala-Cys-Glu-Arg (EACER)	104	167	Tyr 359	483	499	BAC81420.1
Solanum lycopersicum (tomato)	SlNPF6.3	42–46 Glu-Ala-Val-Glu-Arg (EAVER)	102	164	Tyr 354	478	494	XP_004244572.1
Solanum tuberosum (potato)	StNPF6.3	42–46 Glu-Ala-Val-Glu-Arg (EAVER)	101	164	Tyr 354	478	494	XP_006358355.1
Vitis vinifera (grape)	VvNPF6.3	93–97 Glu-Ala-Cys-Glu-Arg (EACER)	153	216	Tyr 408	532	548	WJZ82426.1

Note: *amino acid sequence of SbNPF6.3 found in the *Salicornia* database (https://www.salicorniadb.org/Home.html (accessed on 20 September 2023)).
